# Medical Preaching

**Published:** 1873-04

**Authors:** 


Medical societies are warned not to ask a certain Western minister to preach for them. He has this text ready: "In his disease Asa sought not to the Lord, but to the physicians. And Asa slept with his fathers."

